# Arabidopsis DXO1‐ and RNMT1‐Mediated m^7^G Capping of mRNA Modulates ABA Response

**DOI:** 10.1111/pce.70547

**Published:** 2026-04-16

**Authors:** Shumin Liang, Shangwei Wu, Chen Xiao, Hailei Zhang, Wuzhen Liu, Kefan Hu, Huijuan Tan, Hongwei Guo, Liming Xiong, Jiansheng Liang, Yiji Xia

**Affiliations:** ^1^ Southen University of Science and Technology of China Shenzhen China; ^2^ Department of Biology Hong Kong Baptist University Hong Kong China; ^3^ College of Biological and Environmental Sciences Zhejiang Wanli University Ningbo China; ^4^ Sate Key Laboratory of Agrobiotechnology Chinese University of Hong Kong Hong Kong China

## Abstract

Eukaryotic mRNA carries an N7‐methylguanosine (m⁷G) 5′ cap that is essential for mRNA stability and function. Although traditionally viewed as a constitutive process, m⁷G capping is increasingly recognized as dynamically regulated to control gene expression in response to environmental stimuli. The recent discovery of NAD‐capped RNAs adds further complexity to gene regulation through RNA capping. In Arabidopsis, DXO1, an enzyme with NAD cap decapping and exonuclease activities, was recently shown to also promote m⁷G capping through its non‐catalytic N‐terminal domain, which activates the methyltransferase RNMT1. Here, we show that DXO1's role in abscisic acid (ABA)response and stress tolerance depends primarily on the non‐catalytic domain's function in m⁷G capping, with minimal contribution from its enzymatic functions. RNMT1 mutants similarly exhibit reduced ABA sensitivity, mirroring *dxo1* phenotypes. Transcriptome analysis reveals that DXO1's and RNMT1's functions in m⁷G capping predominantly regulate genes involved in abiotic stress, defense, light signaling, photosynthesis, and transcription. The enzymatic domain of DXO1 modestly influences the expression of a subset of these m⁷G capping‐regulated genes, suggesting coordinated regulation between its m⁷G capping and enzymatic functions. Overall, our study uncovers an important role for m⁷G capping in gene regulation in response to ABA and environmental stimuli.

## Summary statement

A summary statement highlighting the importance of the work is provided below:

The m⁷G cap is a critical regulatory feature of eukaryotic mRNAs, yet its dynamic regulation in plants remains poorly understood. Here, we demonstrate that DXO1's role in ABA and stress responses depends primarily on this N‐terminal domain, which activates the m⁷G methyltransferase RNMT1, with minimal contribution from DXO1's enzymatic activities. Mutations in RNMT1 phenocopy *dxo1* in ABA sensitivity and stress tolerance, and transcriptomic analyses reveal that the DXO1‐RNMT1 module coordinates genes involved in abiotic stress, defense, light signaling, and photosynthesis. Intriguingly, the catalytic domain of DXO1 influences a subset of these same genes, suggesting cross‐talk between m⁷G capping and NAD decapping pathways. Collectively, our findings establish DXO1 as a central hub linking RNA cap metabolism to environmental stress responses in plants, revealing an unanticipated integration of distinct cap regulatory systems.

## 1 Introduction

The N7‐methylguanosine (m^7^G) cap, first discovered over 50 years ago in mRNAs from animal viruses and subsequently in animal and other eukaryotic cells, is a defining feature of eukaryotic mRNA (Furuichi et al. 1975; Shatkin 1976; Sripati et al. 1976; Wei et al. 1975). In animals, two enzymes catalyze the formation of the cap: the capping enzyme (CE) adds the guanosine cap (the G cap) to the 5′ end of a nascent transcript after synthesis of approximately 30 nucleotides and the RNA cap methyltransferase (RNMT) then converts the G cap into the m^7^G cap (Ramanathan et al. 2016; Shuman 1995). In mammals, a small protein named RNMT‐Activating Mini protein (RAM) associates and activates RNMT and is required for efficient cap methylation (Gonatopoulos‐Pournatzis et al. 2011). In addition, after the formation of the m^7^G cap, which is also termed the Cap‐0 structure, the ribose residue on the first nucleotide adjacent to the m^7^G cap typically undergoes 2′‐O‐methylation to form the Cap‐1 structure. mRNA that contains only the Cap‐0 structure is recognized as a non‐self‐molecule to trigger autoimmunity (Daffis et al. 2010; Despic and Jaffrey 2023). As a high level of Cap‐1 mRNA can still cause autoimmunity, a large portion of Cap‐1 mRNAs further undergo 2′‐O–methylation on the second nucleotide in the cytosol to form the Cap‐2 structure (Despic and Jaffrey 2023). In addition to preventing autoimmunity, the Cap‐1 and Cap‐2 structures also improve translation efficiency and mRNA stability (Despic and Jaffrey 2023).

The m⁷G cap plays a critical role in protecting mRNA from degradation by 5′–3′ exonucleases. Additionally, once the cap is formed, it recruits CAP‐BINDING PROTEIN 20 (CBP20), which in turn recruits CBP80 and other factors to assemble the cap‐binding complex. This complex mediates various aspects of mRNA metabolism, including transcriptional elongation, pre‐mRNA processing, mRNA transport, and translation initiation (Flaherty et al. 1997; Izaurralde et al. 1994; Kataoka 2023; Mazza et al. 2001), underscoring the essential role of the cap in mRNA function. While traditionally regarded as a constitutive housekeeping process, growing evidence indicates that m⁷G capping is dynamically regulated in a gene‐specific manner to modulate gene expression in response to stimuli (Borden et al. 2021; Galloway and Cowling 2019). Beyond the canonical m⁷G cap, recent studies have identified non‐canonical RNA caps, such as the NAD cap, present on RNAs in both prokaryotic and eukaryotic organisms including plants (Cahová et al. 2015; Dong et al. 2022; Jiao et al. 2017; Sharma et al. 2022; Walters et al. 2017; Wang et al. 2019; Zhang et al. 2019), further increasing the complexity of RNA capping in gene regulation.

It has been over 40 years since it was reported that plant mRNAs also contain the m^7^G cap (Haugland and Cline 1980; Nichols 1979); however, there has been little studies on the capping process and its regulation in plants till very recently, presumably it was assumed that plants have the same mechanisms in the capping processes. However, recent studies indicate divergence in the m^7^G cap modifications between the animal and plant lineages. Recently, mRNAs in plants and algae have been found to only contain the Cap‐0 structure but lack the Cap‐1 or Cap‐2 structure (Xiao et al. 2025), suggesting distinct mechanisms and function of mRNA capping in gene regulation in plants. Although the capping enzymes and RNMT of Arabidopsis share significant sequence similarities with the animal counterparts (Kerk et al. 2008; Ning et al. 2025; Xiao et al. 2023), it was recently discovered that Arabidopsis RNMT (RNMT1) is activated by DXO1 (Xiao et al. 2023), which was previously reported to be a decapping enzyme for NAD‐capped RNA (NAD‐RNA) (Kwasnik et al. 2019; Pan et al. 2020; Yu et al. 2021). The finding further implies the plant‐specific mechanism of regulation in m^7^G capping and its inter‐connection with NAD decapping.

The DXO family proteins in eukaryotes are known to hydrolyze various RNA caps, including the m^7^G cap, the G cap, and the NAD cap and most of them also possess the 5′–3′ exonuclease activity and are therefore considered as enzymes for RNA quality control (Chang et al. 2012; Doamekpor et al. 2022; Jiao et al. 2017; Jiao et al. 2010; Xiang et al. 2009). Arabidopsis consists of a single DXO protein (DXO1) that does not hydrolyze the G cap or m^7^G cap but possesses the NAD‐RNA decapping (deNADding) activity and a weak exonuclease activity (Kwasnik et al. 2019; Pan et al. 2020). Although the mutation of the mammalian DXO appears not to affect cell growth, the knockout mutation of Arabidopsis *dxo1* leads to pleotropic phenotypes with severe defects in growth, development, fertility, as well as alterations in the ABA inhibition of seed germination and the response to abiotic stresses (Kwasnik et al. 2019; Pan et al. 2020; Yu et al. 2021; Zakrzewska‐Placzek et al. 2025). Surprisingly, the morphological defects of *dxo1* could mostly be complemented by catalytically inactive DXO1 (Kwasnik et al. 2019; Pan et al. 2020), indicating that these phenotypes associated with the *dxo1* mutation are not caused by lacking its enzymatic function but rather by the loss of another critical function of DXO1.

Further studies revealed the interesting discovery that Arabidopsis DXO1 also serves as a key component in m⁷G capping (Xiao et al. 2023). Plant DXO1 proteins are unique in containing a ~ 200 amino acid plant‐specific, non‐catalytic N‐terminal extension (NTE) (Kwasnik et al. 2019; Pan et al. 2020). This NTE interacts with and activates RNMT1, thereby promoting the formation of the m⁷G cap (Xiao et al. 2023). An Arabidopsis RNMT1 knockout mutation was found to be embryonic lethal, while weaker loss‐of‐function alleles (*rnmt1‐2* and *rnmt1‐3*) exhibit growth and developmental defects similar to those observed in *dxo1* mutants. These findings suggest that the pleiotropic phenotypes caused by *dxo1* mutations are due to defects in m⁷G capping, underscoring the importance of the cooperative role of DXO1 and RNMT1 in this process.

Although the ABA‐insensitive phenotype in *dxo1* was previously reported to be attributed to altered NAD‐capped transcriptomes (Yu et al. 2021), our recent finding that DXO1 is also an essential component of the m^7^G capping machinery promoted us to investigate whether it is DXO1's role in deNADding or m^7^G capping that mediates the ABA response. In this report, we present multi‐layered evidence demonstrating that the DXO1's role in the ABA response is attributed to its function in m^7^G capping, not NAD decapping or other catalytic activities. Similarly, DXO1's function in m^7^G capping also contributes to its role in tolerance to drought and salt stresses. Besides, we found that the *rnmt1* mutation leads to similar defects in these responses to those of the *dxo1* mutation. Transcriptome profiling analyses further reveals a critical role of m^7^G capping in mediating ABA and stress responses in plants.

## 2 Results

### 2.1 The DXO1's NAD‐RNA Decapping Activity Does not Confer the ABA Response

The previous report suggests that the reduced ABA sensitivity observed in the *dxo1* mutant might result from a defect in deNADding (Yu et al. 2021). However, our recent discovery that DXO1 also plays an essential role in m^7^G capping through its plant‐specific non‐catalytic N‐terminal extension (NTE) (Xiao et al. 2023) prompted further investigation to distinguish the contributions of NAD decapping and m^7^G capping to the ABA response. To address this, we generated various transgenic lines expressing different DXO1 domains or forms in the *dxo1* mutant background. These included *dxo1* lines expressing the non‐catalytic NTE region (amino acids 1–200), the catalytic C‐terminal domain (cDXO1; amino acids 201–544), and two DXO1 mutants, K412Q and E394A, which were previously found to be catalytically inactive (Pan et al. 2020).

**Figure 1:**
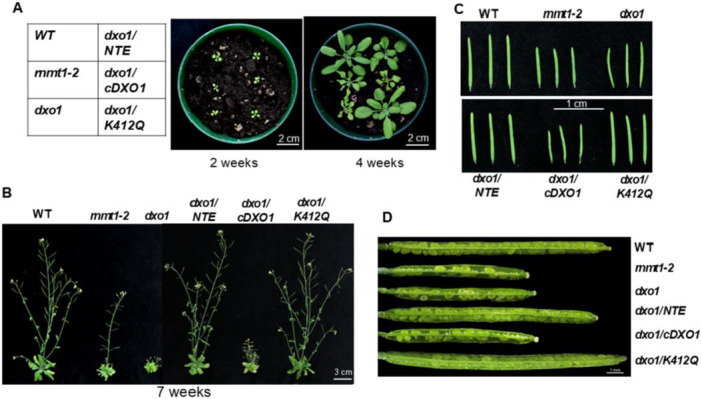
Morphological and developmental phenotypes of WT, *dxo1*, *rnmt1‐2*, and *dxo1* lines expressing different forms of DXO1. (A, B) 2‐week‐ and 4‐week‐old seedlings (A) and 7‐week‐old adult plants of the indicated genotypes. (C, D) Siliques from the different genotypes, showing reduced silique size associated with decreased fertility caused by the *dxo1* and *rnmt1‐2* mutations. *dxo1*/*NTE*, *dxo1*/*cDXO1*, and *dxo1*/*K412Q* refer to *dxo1* lines expressing the N‐terminal extension (NTE), the catalytic domain (cDXO1), and the K412Q mutant form of DXO1, respectively.

The *dxo1* mutant shows no obvious phenotype during seed germination and the early days of seedling growth. However, as the plants mature, they exhibit pleiotropic morphological and developmental defects, including growth retardation, small pale leaves, and partial infertility (Figure 1) (Pan et al. 2020). The *dxo1* phenotypes were fully complemented by the catalytically inactive mutants K412Q and E394A and largely complemented by the NTE domain (Figure 1 and Figure S1). In contrast, the catalytic C‐terminal domain (cDXO1) failed to rescue any of these *dxo1* phenotypes. These results indicate that the morphological and infertility defects in *dxo1* are not due to impaired deNADding or other enzymatic activities but rather depend on the function of its NTE. Furthermore, the partial loss‐of‐function mutant of the RNA cap methyltransferase RNMT1 (*rnmt1‐2* and *rnmt1‐3*) exhibits developmental and morphological phenotypes similar to those of *dxo1* (Figure 1 and Figure S1). *rnmt1‐2* and *rnmt1‐3* are two weak alleles generated by CRISPR/cas‐9 with similar phenotypes whereas the *rnmt1‐1* full knockout mutation is lethal (Xiao et al. 2023).

**Figure 2:**
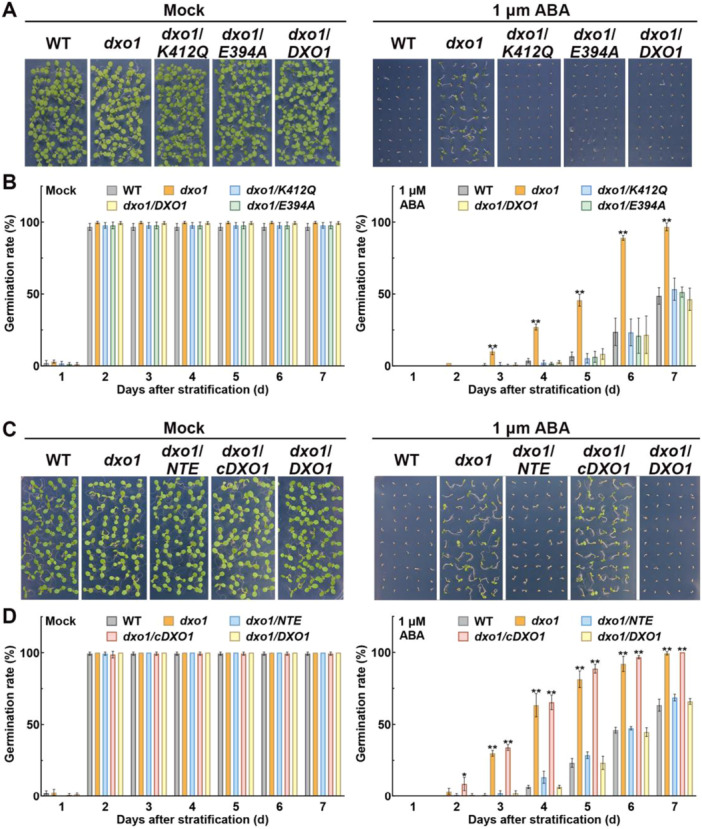
DXO1‐mediated ABA response depends on its non‐catalytic function rather than its catalytic function. (A) Seed germination and seedling phenotypes of WT (Col‐0), *dxo1*, and *dxo1* lines expressing *K412Q*, *E394A*, and the WT *DXO1* genomic copy (*dxo1*/*DXO1*) grown on ½ MS medium without (mock) or with 1 μM ABA for 7 days. (B) Germination rates at indicated days for the genotypes shown in (A), without and with 1 μM ABA. Data represent means ± SD (*n* = 3) with 50 seeds per genotype per replicate. Significant differences relative to WT at each time point were determined by Student's *t*‐test: ** indicates *p* < 0.01. Results are representative of three independent experiments. (C) Seed germination and seedling phenotypes of WT, *dxo1*, and *dxo1* lines expressing the N‐terminal extension (*dxo1*/*NTE*), the catalytic domain (*dxo1*/*cDXO1*), and the full‐length DXO1 (*dxo1*/*DXO1*) grown on ½ MS medium without or with 1 μM ABA for 7 days. (D) Germination rates at indicated days for the genotypes shown in (C), without and with 1 μM ABA. Data represent means ± SD (*n* = 3) with 50 seeds per genotype per replicate. Significant differences relative to WT at each time point were determined by Student's *t*‐test: ** indicates *p* < 0.01. Results are representative of three independent experiments. [Color figure can be viewed at wileyonlinelibrary.com]

We then examined the ABA response phenotypes of the various genotypes. Consistent with the previous report (Yu et al. 2021), the *dxo1* mutant exhibited reduced sensitivity to ABA‐mediated inhibition of seed germination compared to wild type (WT) (Figures S2A,B). Similarly, *dxo1* seedlings showed decreased sensitivity to ABA‐induced growth inhibition relative to WT (Figures S2C,D). To further investigate the role of DXO1's distinct functions in the ABA response, *dxo1* transgenic lines expressing the different DXO1 domains/forms, along with the *rnmt1‐3* mutant, were compared to WT and *dxo1*. Under the mock condition (no ABA), all genotypes exhibited similar germination rates (Figure 2A). However, in the presence of ABA, while *dxo1* seeds germinated at significantly higher rates than WT, the catalytically inactive mutants K412Q and E394A fully complemented this reduced ABA sensitivity phenotype, with *dxo1*/*K412Q* and *dxo1*/*E394A* showing germination rates comparable to WT (Figure 2A,B). These results indicate that the reduced sensitivity to ABA‐mediated germination inhibition in *dxo1* is not due to the defect in DXO1's enzymatic activity.

### 2.2 The N‐Terminal Extension of DXO1, but not Its Catalytic Domain, Is Involved in Its Function in the ABA Response

The C‐terminal catalytic domain Arabidopsis DXO1 is conserved with animal and yeast DXO family proteins. However, Arabidopsis DXO1 contains a ~ 200 amino acid N‐terminal extension (NTE) that is plant‐specific. This NTE is essential and sufficient for interacting with and activating RNMT1 for m^7^G capping (Xiao et al. 2023). Although the complementation of the *dxo1* ABA response phenotype by the catalytically inactive mutants K412Q and E394A (Figure 2A,B) suggests that DXO1's enzymatic activity is not involved in the ABA response, we cannot fully exclude the possibility that these DXO1 mutant forms retain some unknown or undetected enzymatic function. To further clarify the roles of DXO1's enzymatic activity and m^7^G capping in the ABA response, we generated additional *dxo1* lines expressing either the NTE alone or the C‐terminal catalytic domain (cDXO1) and compared their responses to ABA during seed germination and early seedling growth. In the absence of ABA, all genotypes showed germination rates similar to WT and other *dxo1* lines (Figure 2C,D). However, under ABA treatment, *dxo1* and *dxo1*/*cDXO1* exhibited similarly reduced ABA sensitivity with elevated germination rates compared to WT (Figure 2C,D), indicating that the catalytic domain could not complement the ABA response phenotype. In contrast, NTE, like the K412Q and E394A mutants, fully complemented the reduced ABA sensitivity phenotype of *dxo1*, as these lines displayed germination and seedling growth comparable to WT (Figure 2C,D). Together, these findings demonstrate that DXO1's role in the ABA response depends on the noncatalytic NTE domain rather than its enzymatic activity.

### 2.3 The Defect in m^7^G Capping Caused by the *rnmt1* Mutation also Leads to Reduced ABA‐Insensitivity

**Figure 3:**
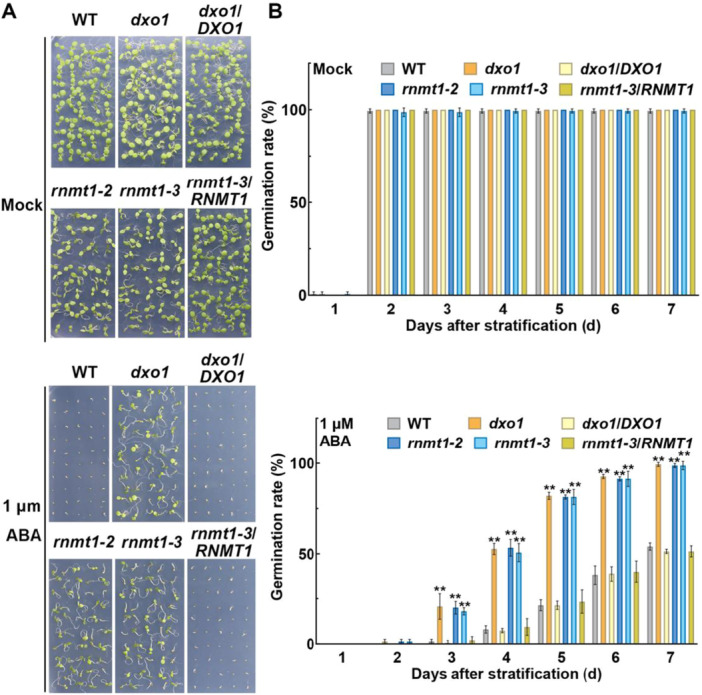
The *rnmt1* mutations confer reduced ABA sensitivity during seed germination and early seedling growth. (A) Seed germination and seedling growth phenotypes of WT, *dxo1*, *dxo1*/*DXO1*, *rnmt1‐2*, *rnmt1*‐*3*, and *rnmt1‐3*/*RNMT1* grown on medium with or without 1 μM ABA for 7 days. Both *dxo1* and *rnmt1* mutants exhibited similar reduced ABA sensitivity phenotypes. (B) Germination rates at indicated days of germination for the genotypes shown in (A). Data represent mean ± SD (*n* = 3) with 50 seeds per genotype per replicate. Significant differences relative to WT at each time point were determined by Student's *t*‐test: ** indicates *p* < 0.01. Results are representative of three independent experiments. [Color figure can be viewed at wileyonlinelibrary.com]

Our finding indicates that the ABA response phenotype caused by the *dxo1* mutations is due to its defect in m^7^G capping. This prompted us to investigate whether the *rnmt1* mutation similarly affects the ABA response. Since the *rnmt1* knockout mutation is embryonic lethal, the two knockdown alleles (*rnmt1‐2* and *rnmt1‐3*) were included in the assay. These *rnmt1* knockdown mutants exhibited morphological phenotypes similar to those of *dxo1* (Figure 1 and Figure S1). Like *dxo1*, both *rnmt1‐2* and *rnmt1‐3* showed reduced sensitivity to ABA‐inhibited germination and seedling growth, whereas the *rnmt1‐3* line complemented with a genomic RNMT1 clone (*rnmt1‐3*/*RNMT1*) displayed phenotypes comparable to WT (Figure 3A,B). These results further demonstrate that defects in m^7^G capping lead to reduced ABA sensitivity, highlighting a critical role for m^7^G capping in ABA response.

### 2.4 The Role of m^7^G Capping in Other Stress Responses

**Figure 4:**
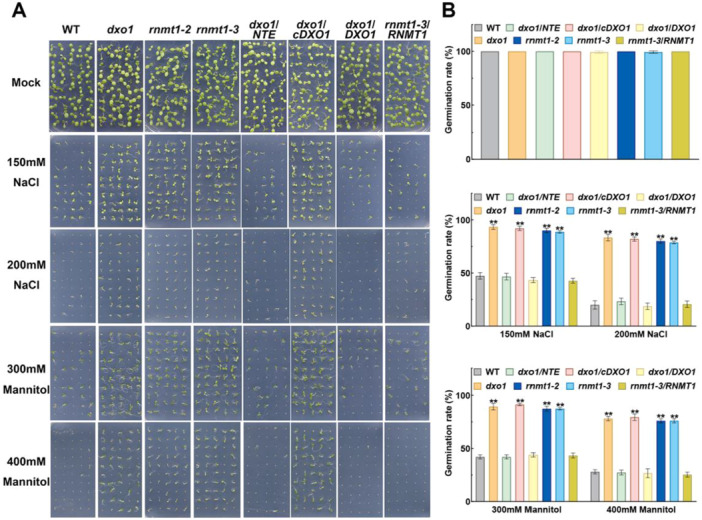
The defect in m^7^G capping caused by *dxo1* and *rnmt1* mutations affect tolerance to high salt and osmotic stresses. (A) Seed germination and early seedling growth of WT and the different *dxo1* and *rnmt1* lines grown with or without 150 mM NaCl, 200 mM NaCl, 300 mM Mannitol, or 400 mM Mannitol for 7 days. (B) Germination rates from (a) were calculated 7 days after stratification under each condition. Data represent mean ± SD (*n* = 3), with 50 seeds per genotype per replicate. Significant differences relative to WT were determined by Student's *t*‐test: * indicates *p* < 0.01. Results are representative of three independent experiments. [Color figure can be viewed at wileyonlinelibrary.com]

Given that ABA is broadly involved in responses to various abiotic stresses, we further investigated the effects of *dxo1* and *rnmt1* mutations on plant stress tolerance to better understand their functional roles in abiotic stress responses. We assessed the responses of different genotypes to various abiotic stresses including salt stress (NaCl treatment), osmotic stress (mannitol treatment), and oxidative stress (methyl viologen treatment) during seed germination and early seedling growth. Under NaCl and mannitol treatments, *dxo1*, *dxo1*/*cDXO1*, and *rnmt1* mutants exhibited higher germination rates compared to WT and other genotypes (Figure 4A,B), indicating that defects in m^7^G capping reduce sensitivity to inhibition of germination and seedling growth by salt and osmotic stresses, although these differences were less pronounced than those observed in response to ABA. We also evaluated heat stress tolerance among these genotypes. As shown in Figure S3A,B, *dxo1*, *dxo1*/*cDXO1*, and *rnmt1* mutants displayed higher survival rates under the stress than WT and *dxo1*/*NTE*, suggesting that impaired m^7^G capping enhances heat stress tolerance. However, the differences among the genotypes in seed germination and early seedling growth under the treatment with methyl viologen (MV) were not obvious (Figure S3C). MV is an agent that induces inhibition of the photosynthetic electron transport chain and production of reactive oxygen species in chloroplasts. These results indicate m^7^G capping plays a role in response to some abiotic stresses.

We quantified overall m⁷G‐capped RNA levels in samples from *dxo1*/*NTE* and *dxo1*/*cDXO1* plants using RNA dot blotting with an anti‐m⁷G antibody (Figure S4). Our results show that m⁷G cap levels were reduced in *dxo1* and *dxo1*/*cDXO1* compared to WT plants. In contrast, *dxo1*/*NTE* plants partially restored m⁷G cap levels, although these levels remained slightly lower than in WT.

### 2.5 The Effect of Defective m^7^G Capping on Overall Transcriptome Profiles With and Without ABA Treatment

**Figure 5:**
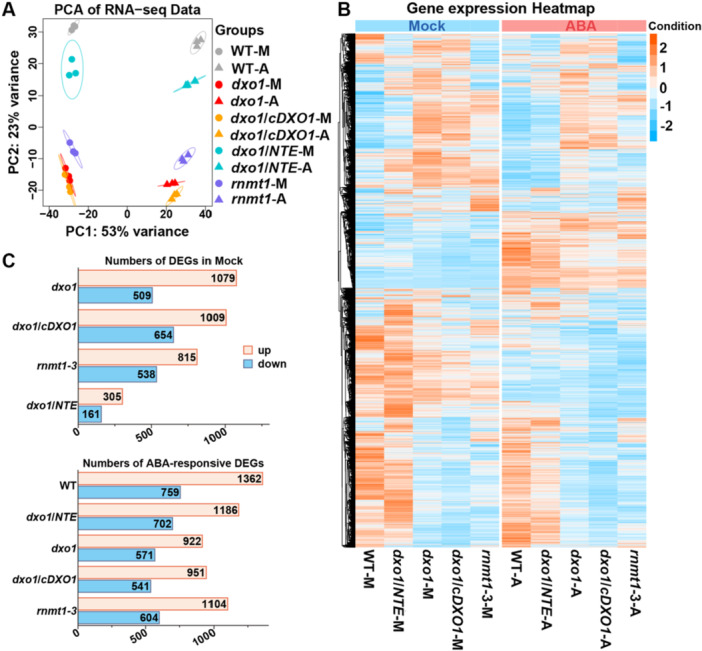
Overview of transcriptome analysis. (A) Principal Component Analysis (PCA) of all samples demonstrating data quality and clustering of transcriptomes by genotype and treatment. (B) Heatmap depicting differential gene expression patterns across genotypes under mock and ABA treatments. M, mock condition; A, ABA condition. The color scale indicates gene regulation direction, from up‐regulation (red) to down‐regulation (blue). (C) Numbers of up‐ and down‐regulated differentially expressed genes (DEGs). The upper panel displays DEGs identified in each genotype compared to WT under mock condition. The lower panel shows DEGs between ABA‐treated and control conditions within each genotype. [Color figure can be viewed at wileyonlinelibrary.com]

To better understand how altered m^7^G capping and other functions of DXO1 affect gene expression, we performed RNA‐seq analysis on 5‐day‐old seedlings of *dxo1*, *dxo1*/*NTE*, *dxo1*/*cDXO1*, *rnmt1‐3* and WT under mock and ABA treatment (50 μM ABA for 3 h). This yielded 10 groups, each with three biological replicates, totaling 30 samples. To assess reproducibility among replicates, Principal Component Analysis (PCA) was conducted on the transcriptome data (Figure 5A). The PCA plot showed tight clustering of replicates within each group, indicating high reproducibility suitable for downstream analysis. Furthermore, the 10 groups were distinctly separated by both ABA treatment and genotypes, reflecting distinct overall transcriptome profiles. The variation in transcriptomes attributable to ABA treatment and genotype was primarily explained by PC1 (53% variance) and PC2 (23% variance). PCA further revealed that the transcriptomes of the *dxo1*/*NTE* cluster closely but remain distinct from WT, suggesting that the NTE largely rescues the transcriptomic effects of the *dxo1* mutation, but the catalytic domain of DXO1 still influences the expression of certain genes. Conversely, the transcriptomes of *dxo1*, *dxo1*/*cDXO1*, and *rnmt1‐3*, each of which is defective in m^7^G capping, show considerable similarity.

Upon ABA treatment, transcriptomic differences between mock and ABA‐treated samples are more pronounced in WT and *dxo1/NTE* compared to *dxo1*, *rnmt1‐3*, and *dxo1/cDXO1* (Figure 5A). These results suggest that defects in m⁷G capping attenuate the global transcriptomic response to ABA. Furthermore, the effect of the *dxo1* mutation on gene expression appears to be primarily driven by altered m⁷G capping, with a much smaller contribution from DXO1's catalytic activity. Nevertheless, some transcriptomic differences persist among *dxo1*, *rnmt1‐3*, and *dxo1/cDXO1*, all of which are defective in m⁷G capping. This is expected, as DXO1 has functions beyond m⁷G capping, and the *dxo1/cDXO1* line retains enzymatic activity. Additionally, although all mutations disrupt overall m⁷G capping, they may differentially affect some specific transcripts.

Heatmap visualization of TPM‐normalized gene expression across all groups revealed distinct transcriptional landscapes between WT and mutant genotypes (Figure 5B). To systematically characterize these differences, we conducted comparative transcriptomic analyses under both mock and ABA treatment conditions. Differentially expressed genes (DEGs) were identified in each mutant relative to WT using DESeq. 2, with significance thresholds set at *p *< 0.05 and at least two‐fold difference (|log2 fold change | > 1) (Figure 5C, Supplementary Table S1). The overall number of DEGs in *dxo1*, *dxo1*/*cDXO1*, and *rnmt1‐3* mutants was comparable under both conditions, whereas *dxo1*/*NTE* exhibited notably fewer DEGs relative to WT. Additionally, transcriptome profile differences among *dxo1*, *dxo1*/*cDXO1*, and *rnmt1‐3* were small in both treatment groups (Figure 5B).

### 2.6 Transcriptomic Impact of Defective m⁷G Capping Under Mock Condition

To investigate the effect of defective m⁷G capping on transcriptomes, we performed functional analyses of DEGs in *dxo1*/*cDXO1* and *rnmt1‐3* mutants compared with WT, two genotypes both impaired in m⁷G capping. We first examined gene expression under normal conditions (without ABA treatment). Gene Ontology (GO) enrichment analysis was conducted on 1,663 DEGs in *dxo1*/*cDXO1* and 1,353 DEGs in *rnmt1‐3* (Table S2). Overall, GO enrichment patterns across biological processes (BP), cellular compartments (CC), and molecular functions (MF) were similar between the two genotypes.

**Figure 6:**
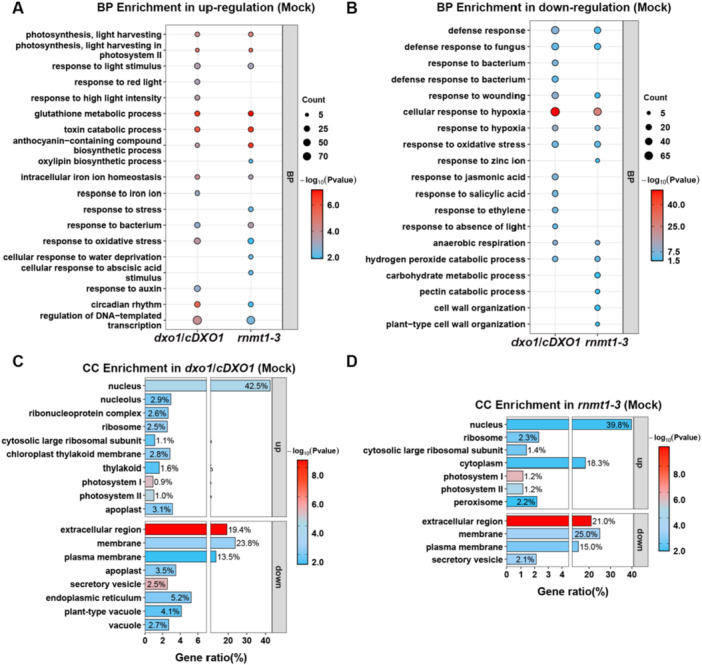
Functional categories of DEGs in *dxo1*/*cDXO1* and *rnmt1‐3* in comparison with WT. (A) Biological processes enriched among up‐regulated DEGs in *dxo1*/*cDXO1* and *rnmt1‐3*. (B) Biological processes enriched among down‐regulated DEGs in *dxo1*/*cDXO1* and *rnmt1‐3*. (C, D) Cellular compartments enriched among DEGs in *dxo1*/*cDXO1* (C) and *rnmt1‐3* (D). BP, Biological Process; CC, Cellular Compartment. [Color figure can be viewed at wileyonlinelibrary.com]

Upregulated DEGs in both mutants were significantly enriched in GO terms related to stress tolerance, including glutathione metabolism, anthocyanin biosynthesis, toxin catabolism, and responses to oxidative stress, heat, iron ion homeostasis, and bacterial stimuli (Figure 6A, Table S2). Additional enriched terms among upregulated genes included DNA transcription processes, photosynthesis, and light response (Figure 6A, Table S2). The downregulated DEGs in both mutants were enriched also for responses to abiotic and biotic stresses such as defense response, hypoxia, oxidative stress, and wounding. Notably, *dxo1*/*cDXO1* showed reduced expression of genes involved in responses to jasmonic acid, salicylic acid, ethylene, and absence of light, whereas *rnmt1‐3* exhibited downregulation of genes related to carbohydrate metabolism and cell wall organization (Figure 6B).

Similarly, large overlaps were observed between DEGs in *dxo1*/*cDXO1* and *rnmt1‐3* in MF and CC categories. Molecular function analysis highlighted enrichment of transcription and DNA‐binding activities among DEGs (Figure S5). In the cellular compartment category, upregulated DEGs were predominantly localized to the nucleus (45.4% in *dxo1*/*cDXO1* and 40% in *rnmt1‐3*), consistent with enrichment for “regulation of DNA‐templated transcription”, indicating that defective m⁷G capping prominently affects genes in transcription processes (Figure 6C,D). Additionally, subsets of DEGs encoded proteins localized to chloroplasts (6.3% in *dxo1*/*cDXO1* and 2.3% in *rnmt1‐3*) and ribosomes (3.6% in *dxo1*/*cDXO1* and 3.9% in *rnmt1‐3*) (Figure 6C,D). In contrast, over 50% of downregulated DEGs in both mutants encoded proteins localized to non‐nuclear compartments such as the extracellular region, membrane, and secretory vesicles (Figure 6C,D).

The functional categories of DEGs in the *dxo1* mutant, defective in both m⁷G capping and enzymatic activity, were highly similar to those observed in *dxo1*/*cDXO1* (Figures 5 and Figure S6), supporting the conclusion that impaired m⁷G capping is the primary driver of transcriptomic alterations in *dxo1*.

### 2.7 The Effect of m⁷G Capping on Gene Expression in Response to ABA

**Figure 7:**
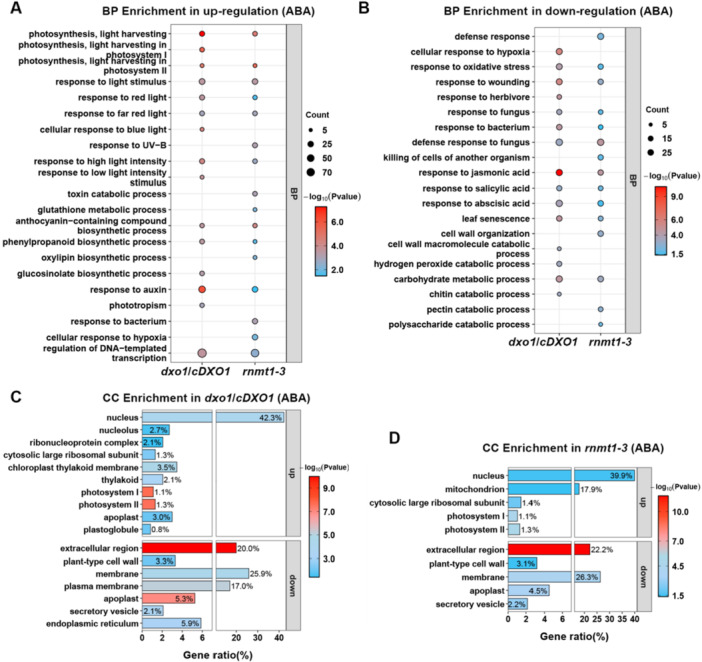
GO enrichment analysis of ABA‐responsive DEGs in *dxo1*/*cDXO1* and *rnmt1‐3* vs. WT. (A, B) Biological processes enriched among up‐regulated (A) and down‐regulated DEGs (B) in *dxo1*/*cDXO1* and *rnmt1‐3*. (C, D) Cellular compartments enriched among DEGs in *dxo1*/*cDXO1* (C) and *rnmt1‐3* (D). The color scale represents log10(Pvalue), ranging from high (red) to low (blue). BP, Biological Process; CC, Cellular Compartment. [Color figure can be viewed at wileyonlinelibrary.com]

We then compared the transcriptomes of different genotypes following ABA treatment. In this study, genes whose expression was significantly induced or repressed by ABA were defined as “ABA‐responsive genes.” In WT plants, we identified 2,121 ABA‐responsive genes; however, this number was reduced in *dxo1* (1,493), *dxo1*/*cDXO1* (1,492), and *rnmt1‐3* (1,708) mutants (Figure 5C). Consistently, heatmap analysis revealed that the transcript levels of ABA‐responsive genes were reduced in these three genotypes compared to WT (Figure 5B). These results indicate that defects in m⁷G capping attenuate the gene expression response to ABA. Furthermore, following ABA treatment, downregulated DEGs in *dxo1*/*cDXO1* and *rnmt1‐3* relative to WT were significantly enriched for the GO term “response to abscisic acid” (Figure 7A, Table S2), which aligns with their observed reduced ABA sensitivity phenotype. Among the genes that are known to be involved in ABA signaling, the genes encoding the ABA receptors PYL1/4/5/6 and DOG1 (DELAY OF GERMINATION 1) exhibited higher expression whereas these coding for other ABA signaling components such as PP2Cs and SnRK2s, MAPK modules (MAPKKK17 and MAPKKK18) and ABA‐responsive proteins RD20 and COR15B displayed lower expression in *dxo1*, *dxo1*/*cDXO1* and *rnmt1‐3* compared with WT (Figure S7), suggesting weak ABA signal transduction in mutants.

Besides, in the up‐regulated genes, light responses and stress response genes, including the genes involved in metabolism of glutathione and anthocyanins, were prominently enriched in *dxo1*/*cDXO1* and *rnmt1‐3* (Figure 7A). Moreover, similar to mock condition, the up‐regulated DEGs in *dxo1*, *dxo1*/*cDXO1* and *rnmt1‐3* are enriched prominently in the nucleus processes whereas the down‐regulated DEGs are mostly in the non‐nuclear compartments (Figure 7C,D). The down‐regulated genes were also enriched in stress responses (e.g. response to fungus and abiotic stresses) as well as in catabolic processes (e.g. carbohydrate metabolic process and chitin catabolic process) related to cell wall construction (Figure 7B). Similar terms were found in ABA‐responsive genes of *dxo1* (Figure S6C,D), further supporting that stress dysregulation contributes to ABA regulation through the m^7^G capping components.

Overall, the transcriptome profiling analysis indicates that the mutations that lead to the defect in m^7^G capping have profound effects on the expression of genes involved in transcriptional processes and responses to environmental stimuli such as abiotic and biotic stress responses and light responses. This establishes m^7^G capping as a key regulator of transcriptomic plasticity, balancing stress resilience, energy metabolism, and developmental progression through gene regulation.

### 2.8 The Effect of DXO1's Catalytic Functions on Gene Expression

**Figure 8:**
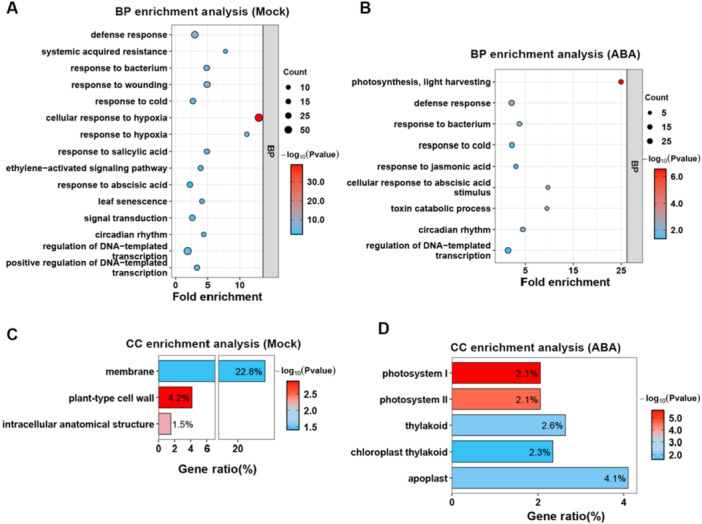
Functional enrichment analysis for DXO1's enzymatic activities. (A, B) Biological process enrichment in mock condition (A) and in response to ABA among the DGEs of *dxo1*/*NTE* vs WT. (C, D) Cellular compartment enrichment DGEs in mock condition (C) in response to ABA (D). The color bar indicates ‐log10(Pvalue) from high (red) to low (blue). BP, Biological Process; CC, Cellular Compartment. [Color figure can be viewed at wileyonlinelibrary.com]

NTE of DXO1 largely rescued the growth and developmental defects of *dxo1* and fully rescued its ABA hyposensitivity phenotypes (Figure 1 and Figure S1). However, there are residual transcriptomic differences between *dxo1*/*NTE* and wild‐type (WT) plants, suggesting that DXO1's catalytic activities, such as deNADding, contribute to regulation of some genes. To dissect the role of its enzymatic functions, *dxo1*/*NTE* and WT transcriptomes were compared, which led to the identification of 466 and 360 DEGs under mock and ABA conditions, respectively, between these two genotypes (Figure 5C, Supporting Table S1). The numbers of the DEGs are much smaller than DGEs caused by the m^7^G defect mutants; however, these DEGs are also enriched in the functional categories of responses to abiotic and biotic stresses (Figure 8A), which are overlapped to those in the m^7^G capping‐deficient mutants. In response to the ABA treatment (Figure 8B), the ABA‐responsive DEGs of *dxo1*/*NTE* enriched mostly also in photosynthesis and abiotic and biotic responses. Strikingly, the majority of genes with altered expression in *dxo1/NTE* compared to WT also showed differential expression in *dxo1/cDXO1*: 59% (275/466) of the DEGs under mock conditions and 67.5% (243/360) of the ABA‐responsive DEGs in *dxo1/NTE* overlapped with those in *dxo1*/*cDXO1* (Figure S8A,B). This significant overlap indicates that the enzymatic functions of DXO1 also influence the expression of genes regulated by m⁷G capping, albeit to a more moderate extent. These findings suggest a coordinated interplay between DXO1's enzymatic activities and m⁷G capping in co‐regulating many stress‐ and light‐responsive genes.

## 3 Discussion

### 3.1 The Unique Mechanism of m⁷G Capping Activation in Plants

It has been known for over 40 years that mRNAs in plants carry the m^7^G cap (Haugland and Cline 1980; Nichols 1979). However, the processes and regulatory mechanisms underlying m^7^G capping have been minimally studied until recently, likely due to the presumption that they mirror those in yeast and animals. The enzymes responsible for the formation of the m^7^G cap have only recently been identified and characterized. Although the Arabidopsis capping enzyme and RNMT exhibit high sequence similarity to their mammalian counterparts (Kerk et al. 2008; Ning et al. 2025; Xiao et al. 2023), it appears that animals and plants have evolved distinct mechanisms for the activation of m^7^G capping. In mammals, RNMT is activated by the mini protein RAM, which has no other known functions. In contrast, Arabidopsis RNMT1 is activated by DXO1 (Xiao et al. 2023), which also possesses deNADding and exonuclease activities (Kwasnik et al. 2019; Pan et al. 2020). The plant DXO1 features approximately 200 amino acid plant‐specific N‐terminal extension (NTE) that is both essential and sufficient for activating RNMT1. These findings suggest that the regulation of m^7^G capping in plants involves unique mechanisms and raises intriguing questions about the potential interactions between m^7^G capping and NAD decapping mediated by DXO1. Furthermore, plant DXO1 is unable to decap the m7G cap or the G cap, whereas DXO family proteins in animals typically possess the ability to hydrolyze the G cap and/or m^7^G cap, but have no reported role in m^7^G capping.

### 3.2 The DXO1's Role in m7G Capping through its Non‐Catalytic N‐Terminal Extension is Central to Plant Development and ABA Response

Previous reports indicated that DXO1 plays a role in the ABA response, presumably through its deNADding function (Yu et al. 2021). However, our study presents multiple lines of evidence demonstrating that it is DXO1's non‐catalytic NTE, rather than its catalytic domain, that is responsible for the observed reduced ABA‐response phenotype. Furthermore, the diverse morphological and developmental phenotypes associated with the *dxo1* mutant can be nearly fully complemented by the NTE of DXO1, consistent with previous findings that a catalytically inactive DXO1 mutant, but not its catalytic domain alone, can also complement these phenotypes (Kwasnik et al. 2019; Pan et al. 2020). Our RNA‐seq data further reveal that the transcriptomes of *dxo1* complemented with the NTE of DXO1 closely resemble that of wild type (WT), while the transcriptome of *dxo1* complemented with its catalytic domain resembles that of *dxo1* under both mock conditions and ABA treatment. This further underscores the critical role of the NTE of DXO1, not its enzymatic function, in these developmental and physiological processes, including the ABA response.

The long NTE of DXO1 is predicted to be largely intrinsically disordered, which might be involved in protein‐protein interactions or interaction with RNA/DNA. In addition to binding to and activating RNMT1, the NTE likely interacts with other proteins, as indicated by our previous yeast two‐hybrid screen (Xiao et al. 2023). Therefore, NTE may have additional functions beyond m^7^G capping that have yet to be defined. Our findings show that the *rnmt1* mutants displays phenotypes similar to those of *dxo1* and *dxo1* complemented with the DXO1 catalytic domain, including the ABA response phenotype and transcriptomic profiles with and without ABA treatment. This strongly suggests that the developmental and ABA response phenotypes observed in *dxo1* are indeed attributed to DXO1's role in m^7^G capping.

### 3.3 The Roles of m⁷G Capping in ABA Response and Other Plant Biological Processes

Upon the formation of the m^7^G cap during the early stage of transcription, it is recognized and bound by CAP BINDING PROTEIN 20 (CBP20), which subsequently associates with CBP80 to form the cap‐binding complex (CBC). The CBC then recruits processing factors that mediate transcriptional elongation, splicing, mRNA transport, and translation (Gonatopoulos‐Pournatzis and Cowling 2014). Mutations in CBP20 and CBP80 in plants have been shown to alter responses to ABA and various abiotic stresses. A mutation in the Cap Binding Protein 20 gene confers drought tolerance (Bacsó and Papp 2008; Papp et al. 2004). The CBP80 protein, also called ABH1 (*ABA HYPERSENSITIVE MUTANT 1*), modulates early abscisic acid signal transduction in Arabidopsis (Hugouvieux et al. 2001). It has also been reported that CBP20 and CBP80 are involved in processing of primary microRNAs and ethylene‐mediated phosphorylation of CBP20 is linked to microRNA production and root growth (Kim et al. 2008; Zhang et al. 2016). Mutations in CBP20 and CBP80 both affect splicing of many pre‐mRNAs (Raczynska et al. 2010). Interestingly, other than the ABA hypersensitivity phenotype, *abh1* does not exhibit significant alterations to all other tested hormones and only displays very mild growth and developmental phenotypes (Hugouvieux et al. 2001). These findings suggest that the regulation of m7G capping may play a more critical role in the ABA response compared to other physiological and developmental processes.

### 3.4 Transcriptomic Insights Into the Coordinated Regulatory Network

Transcriptomic analysis reveals that genes whose expression is altered by defects in m^7^G capping, such as those observed in the *dxo1*, *dxo1/cDXO1*, and *rnmt1* mutants, are predominantly enriched in pathways related to abiotic and biotic stress responses, as well as light response and photosynthesis with or without ABA treatment. The differential expression of numerous stress‐response genes provides a molecular basis for the ABA‐response and abiotic stress tolerance phenotypes associated with m^7^G capping defects. Notably, most genes affected by mutations impairing DXO1's catalytic activity (*dxo1/NTE*) overlap with those altered by m^7^G defects, albeit to a lesser extent, suggesting that DXO1's catalytic activities, such as the deNADding enzyme or exonuclease, contribute to regulation of some genes. A recent study reported that both the NTE and cDXO1 domains contribute to immune responses, with the cDXO1 domain playing a minor role (Golisz‐Mocydlarz et al. 2025). These findings suggest a coordinated regulatory role of m^7^G capping and DXO1's catalytic functions in modulating gene expression.

### 3.5 Unresolved Questions on the Role of DXO1's Enzymatic Function and Future Perspectives

Although NAD‐RNAs have been identified in diverse organisms various organisms (Borden et al. 2021), plants (Dong et al. 2022; Wang et al. 2019; Zhang et al. 2019), their biological and molecular functions remain largely unclear. Our data show that the developmental and physiological defects of the *dxo1* mutant can be complemented not only by catalytically inactive DXO1 but also substantially by the NTE domain alone. This raises important questions regarding the potential functions of NAD‐capped RNAs and the role of DXO1's deNADding activity. Our transcriptome profiling data indicates that mutations impairing DXO1 enzymatic activity also affect genes involved in abiotic and biotic stress responses and light/photosynthesis pathways, implying a possible role for DXO1's deNADding function in these processes.

Once thought to be a constitutive process, growing evidence now indicates that m^7^G capping is dynamically regulated in a gene‐specific manner in response to environmental stimuli (Borden et al. 2021; Cowling 2009). The recent discovery of NAD‐capped RNAs adds an additional layer of complexity to gene regulation mediated by RNA capping. The activation of m^7^G capping by DXO1 in plants underscores the intricate interplay between m^7^G capping and NAD capping/decapping mechanisms in controlling gene expression. Continued investigation in this area promises to further elucidate the sophisticated regulatory networks governed by RNA capping.

## 4 Materials and Methods

### 4.1 Plant Materials and Growth Conditions

The *Arabidopsis thaliana* T‐DNA insertion mutant *dxo1*, *rnmt1‐3*, along with transgenic lines *DXO1pro*::DXO1/*dxo1* (*dxo1*/*DXO1*), *DXO1pro*::*DXO1‐K412Q*/*dxo1* (*dxo1*/*K412Q*), *DXO1pro*::*DXO1‐E394A*/*dxo1* (*dxo1*/*E394A*), *35S*::*DXO1‐FLAG #8* (*OE‐DXO1 #8*), *35S*::*DXO1‐FLAG #9* (*OE‐DXO1 #9*), *RNMT1pro*::*RNMT1*/*rnmt1* (*rnmt1*/*RNMT1*) were previously described (Pan et al. 2020; Xiao et al. 2023). In this study, we generated two additional transgenic lines: *DXO1pro*::*DXO1‐N*/*dxo1* (*dxo1*/*NTE*) and *DXO1pro*::*DXO1‐C*/*dxo1* (*dxo1*/*cDXO1*). All plants were grown on half‐strength Murashige and Skoog (½ MS) medium (1% sucrose, 0.8% agar, pH 5.7–5.8) for experiments and grown in soil for seeds harvest. All plants were placed in a walk‐in growth room under controlled conditions (22°C, 60% relative humidity, 16‐h light/8‐h dark photoperiod).

### 4.2 Plasmid Construction and Transgenic Plant Generation

To generate *DXO1pro*::*DXO1‐N*/*dxo1* (*dxo1*/*NTE*) and *DXO1pro*::*DXO1‐C*/*dxo1* (*dxo1*/*cDXO1*), the DXO1 coding sequence (CDS) was divided into N‐terminal (amino acids 1–200; NTE) and C‐terminal (amino acids 201–544; cDXO1) fragments. These fragments were cloned into a modified pCambia1305 vector (Pan et al. 2020) harboring the 2,004‐bp DXO1 promoter, yielding pCambia1305‐*DXO1pro::DXO1‐N* and pCambia1305‐*DXO1pro::DXO1‐C*. These plasmids were introduced into the *dxo1* mutant via Agrobacterium tumefaciens‐mediated floral dip transformation (Clough and Bent 1998).

### 4.3 Seed Germination Assays

Seeds of the genotypes for comparisons were harvested at the same batches and air‐dried for ≥ 4 weeks before the germination assays. For the germination assays, at least 50 seeds of each genotype were used in each replicate and at least three replicates for each assay to measure the germination rates. After surface‐sterilized, the seeds of each genotype were stratified at 4°C in darkness for 72 h before being sown on half‐strength Murashige and Skoog (½ MS) medium supplemented with or without ABA, NaCl, mannitol or methyl viologen (MV). Seeds were germinated under long‐day conditions (16‐h light/8‐h dark cycles). The emergence of radicle was recognized as seed germinated and the germination rates were recorded at the indicated time points.

### 4.4 Root Elongation Assays

For root elongation assay, 4‐day‐old seedlings grown on the MS medium were transferred to fresh 1/2 MS medium ± 10 μm ABA. And then seedlings were allowed to grow vertically for additional 5 days. Primary root length was measured using ImageJ (Schneider et al. 2012).

### 4.5 Heat Stress Tolerance Assays

To assess thermotolerance, 5‐day‐old seedlings cultivated at 22°C were subjected to acute heat stress (45°C for 45 min) followed by a 72‐h recovery under standard growth conditions (22°C). Three independent biological replicates were performed, with 42 seedlings per genotype per replicate. Survival rates were quantified as the percentage of seedlings exhibiting regrowth after recovery, serving as the primary metric for heat stress tolerance evaluation.

### 4.6 Quantification of m^7^G Cap Levels

Total RNA was extracted from 4‐week‐old plants of WT, *dxo1*, *dxo1*/*NTE*, and *dxo1*/*cDXO1* using TRIzol reagent (Thermo Fisher, Cat. No. 15596026) following the manufacturer's instructions. mRNA was enriched using Oligo d(T)₂₅ magnetic beads (NEB, Cat. No. S1419S). Approximately 200 ng of mRNA from each sample was subjected to RNA dot blotting and probed with an anti‐m⁷G antibody to assess m⁷G cap levels.

### 4.7 RNA Sequencing and Data Analysis

To perform RNA‐seq analysis, approximately 100 mg of 5‐d‐old seedlings of each genotype were treated with or without 50 μM ABA for 3 h (seedlings were transferred to ABA‐containing MS medium), and then sampling for RNA extraction using TRIzol reagent (Thermo Fisher, Cat. No. 15596026) following the manufacturer's instruction. Total RNA was used for mRNA library construction and sequenced on Illumina NovaSeq. 6000 platform (Novogene Incorporation, Beijing, China).

After sequencing, raw reads were subjected to quality control and trimmed by using fastqc (v0.11.8) to get high‐quality reads. High‐quality reads were mapped to the Arabidopsis genome (TAIR10) using hisat2 (v2.2.1). Calculated the read counts of each gene with FeatureCounts (included in Subread v2.0.8 software) to generate the count matrix for expression analysis. The count matrix was normalized to transcripts per million (TPM) for cross‐sample comparisons. And the expression TPM data for some genes were visualized with heatmap.

### 4.8 Differential Expression Analysis

The count matrix was used for differential expression analysis by DESeq. 2 R package according to the pair‐wise comparison listed in supplementary Table S1. And the significant differentially expressed genes (DEGs) were identified with the criteria |foldchange | > 2 (|log2 (FC)| > 1) and *p*‐value < 0.05. Principle component analysis (PCA) was performed on all samples with count matrix using DESeq. 2 to assess the data quality.

### 4.9 Gene Function Annotation

Gene functions were annotated using Ensembl Plant (Table S1). Gene ontology (GO) and KEGG pathway enrichment analyses were performed on DEGs using DAVID (Sherman et al. 2024). Significant terms (*p*‐value < 0.05) were shown in Tables S2, with the most significant terms (*p*‐value < 0.05, gene count ≥ 5) visualized in figures.

## Conflicts of Interest

The authors declare no conflicts of interest.

## Supporting information

Supporting File 1

Supporting File 2

Supporting File 3

## Data Availability

The RNA‐seq data have been submitted to the National Center for Biotechnology Information Gene Expression Omnibus repository, https://www.ncbi.nlm.nih.gov/geo and their accession number is GSE300339.
