# Supplementary material for: Arabidopsis DXO1‐ and RNMT1‐Mediated m7G Capping of mRNA Modulates ABA Response
Source: Plant Cell Environ. 2026 Apr 16;49(8):5358–72. doi: 10.1111/pce.70547 (PMC13353720; doi:10.1111/pce.70547)
Supplement: Supplementary file 1 — Supporting File 1 [file PCE-49-5358-s002.docx]

**Supporting Information**

| **Filename** | **Description** |
| --- | --- |
| **Supplementary_Table S1.xlsx** | **Table S1.** Summary of DEGs |
| **Supplementary_Table S2.xlsx** | **Table S2.** GO Functational Analysis |

**Supplementary Figure**

**
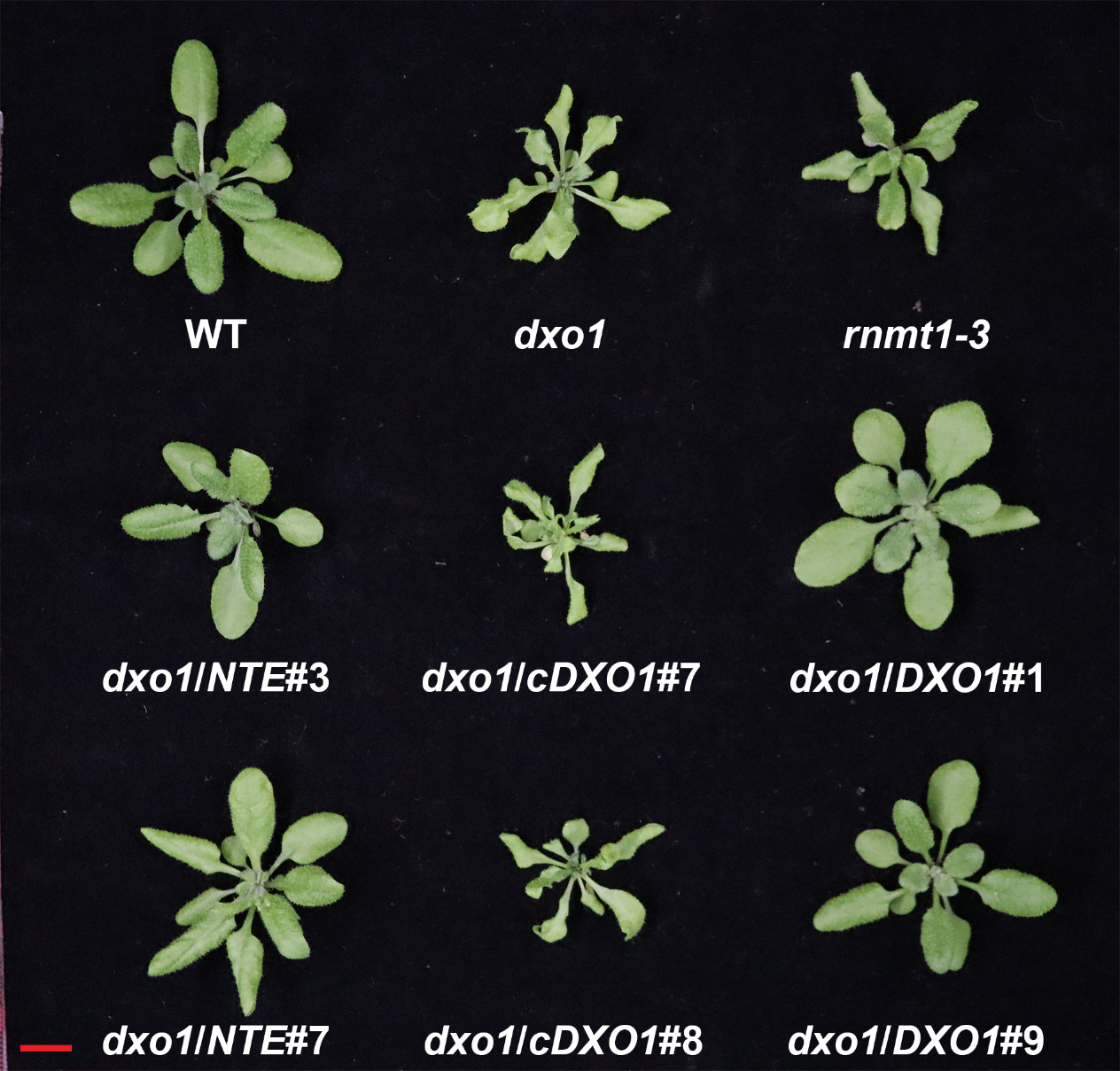
Figure S1. Morphological phenotypes of 4-week-old plants of Arabidopsis genotypes.**

Plants grown in soil include wild-type (Col-0), *dxo1*, *rnmt1*-*3*, multiple *dxo1* lines complemented with the various DXO1 domains: *dxo1*/*NTE* (#3, #7), *dxo1*/*cDXO1* (#7, #8), *dxo1*/*DXO1* (#1, #9). Scale bar = 1▒cm.

**
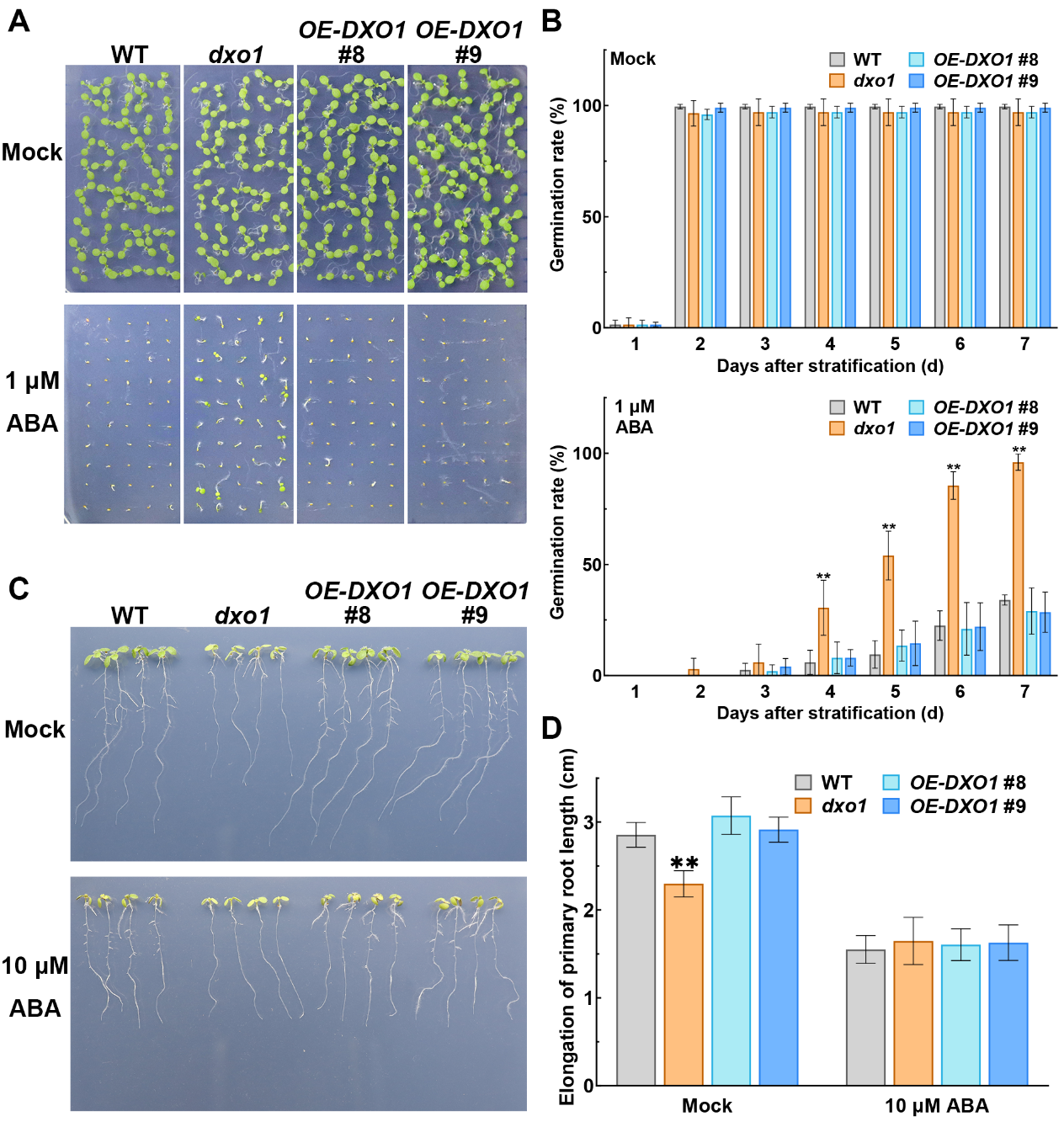
Figure S2. ABA hyposensitivity of *dxo1* during germination and seedling development.**

**(A)** Germination phenotypes of WT, *dxo1*, and DXO1-overexpression (*OE-DXO1* #8, #9) seeds on ½ MS▒±▒1▒μM ABA. Representative images at final time point.

**(B)** Germination rate in **(A)** were recorded over 7 days (mean▒±▒SD, n▒=▒3 replicates, 50 seeds/genotype/replicate).

**(C)** Root growth phenotypes of 4-day-old seedlings transferred to ½ MS▒±▒10▒μM ABA for 5 days.

**(D)** Primary root length quantification (mean▒±▒SD, n▒=▒4 replicates, 4 seedlings/genotype/replicate). Significant differences relative to WT were determined by Student’s *t*-test: ** indicates *p*-value▒<▒0.01.

**
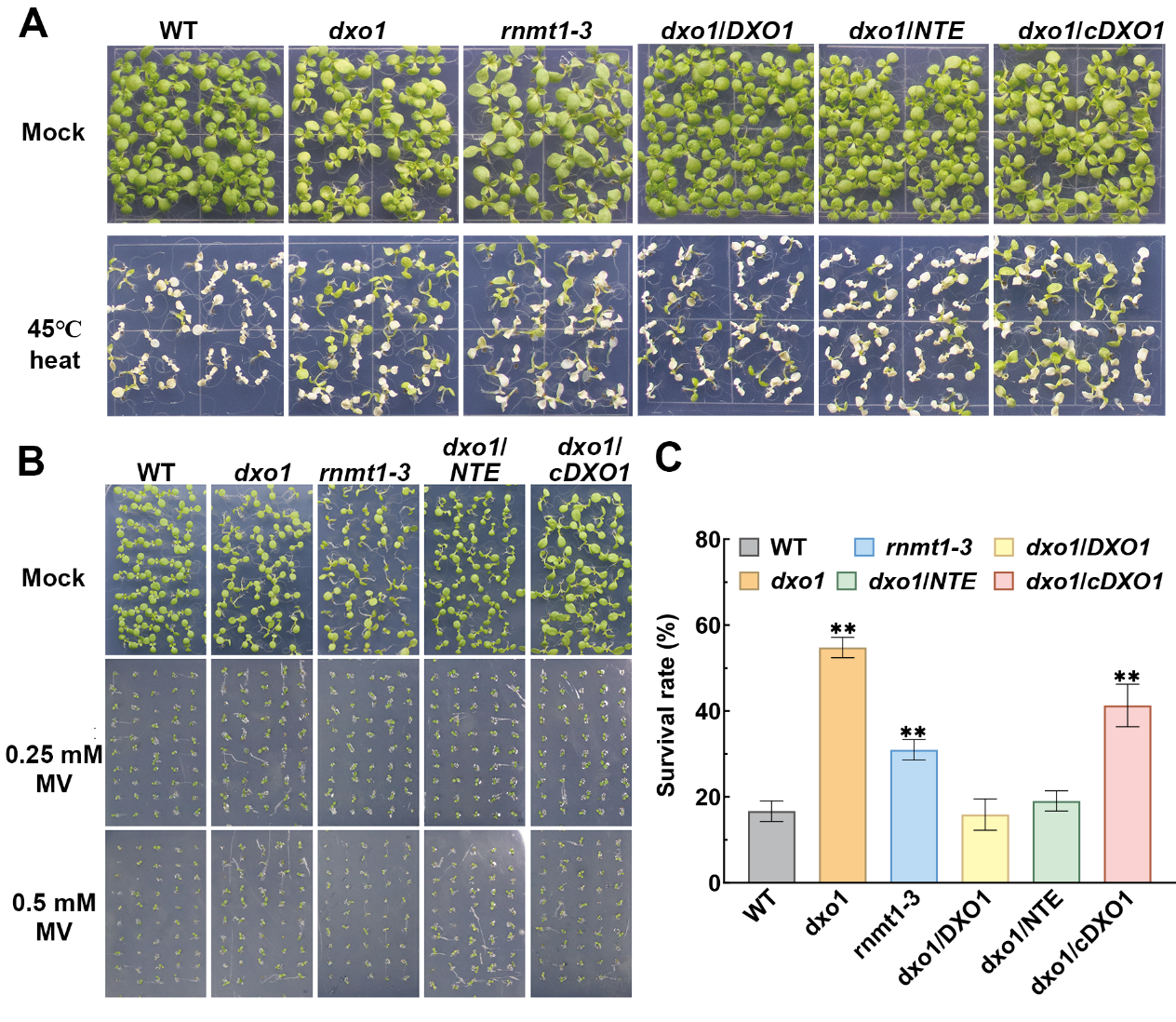
Figure S3. Thermotolerance impairment in *dxo1* and *rnmt1-3* mutants.**

**(A)** Plants of WT, *dxo1*, *rnmt1-3*, *dxo1*/*DXO1*, *dxo1*/*NTE* and *dxo1/cDXO1*, were treated with and without heat (45°C) for 45▒min, then recover under 22°C for 3 days to measure the survival rate **(C)**.

**(B)** Seeds of WT, *dxo1*, *rnmt1*, *dxo1*/*NTE* and *dxo1*/*cDXO1* were treated with and without 0.25▒mM and 0.5▒mM MV, and No significant differences observed (n▒=▒3 biological replicates). Significant differences relative to WT were determined by Student’s *t*-test: ** indicates *p*-value▒<▒0.01.

**
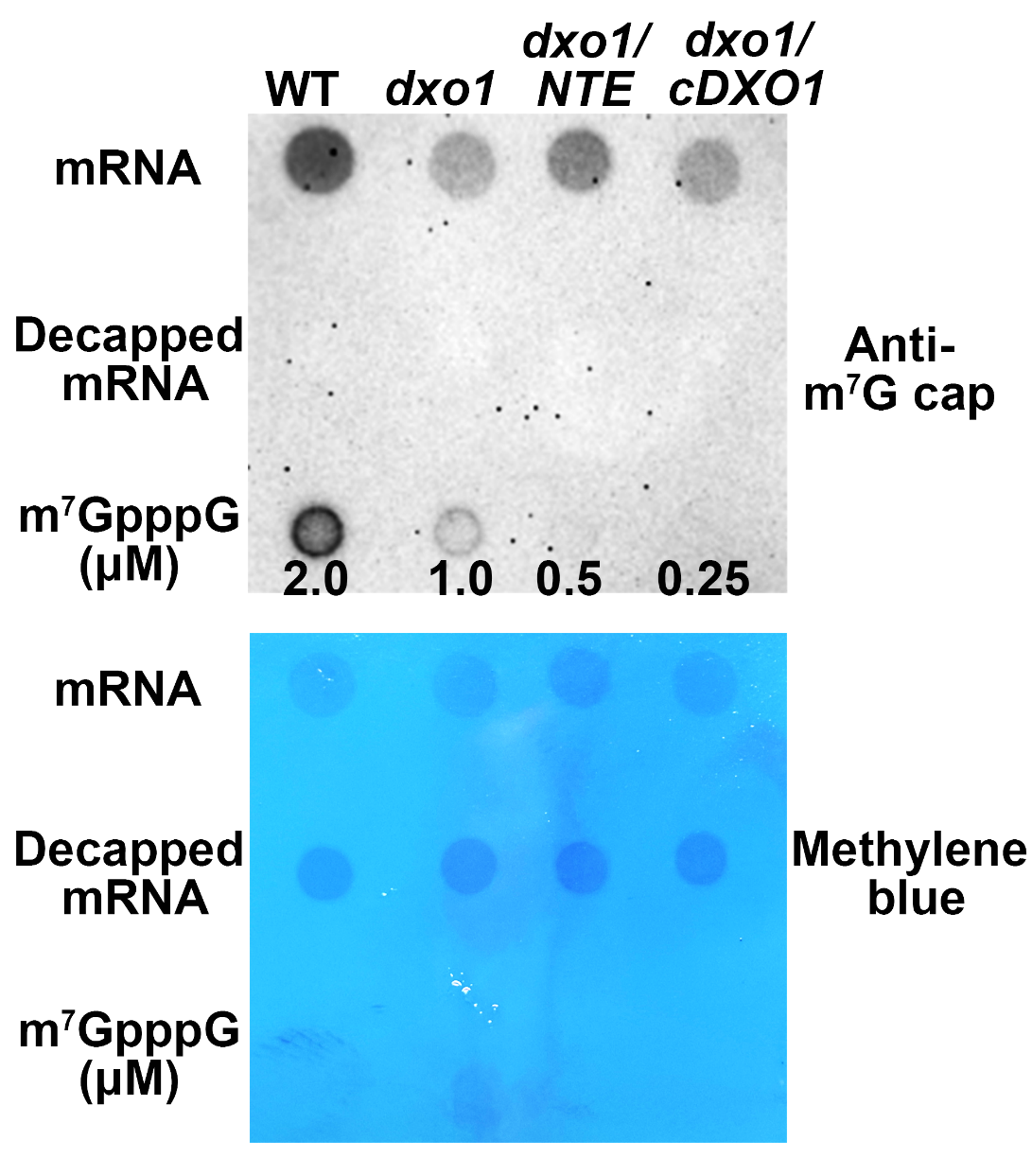
Figure S4. Analysis of m^7^G cap levels in different genotypes.** The m^7^G cap levels in WT, dxo1, dxo1/NTE and dxo1/cDXO1 were analyzed by RNA dot blotting. The same amount of poly(A)-enriched RNAs from each sample was added to the membrane and detected with the anti-m7G antibody. Decapped mRNA (treated with a decapping enzyme (NEB, Cat. No. M0608S)) served as a negative control, and a dilution series of m⁷GpppG was used as a standard for quantification. Three independent experiments were performed with similar results; representative data are shown.


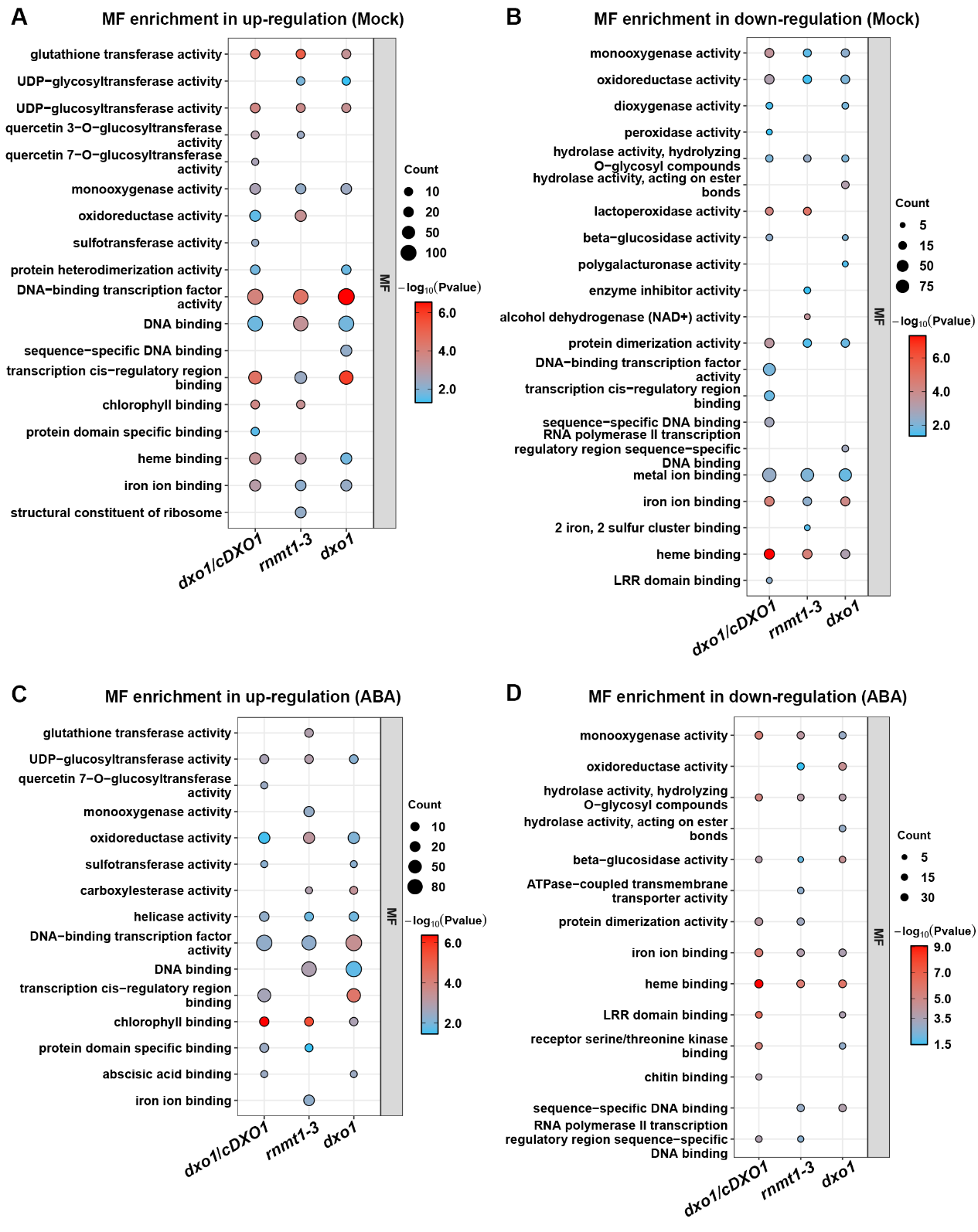
**Figure S5. GO enrichment of MF categories in DEGs of *dxo1*/*cDXO1*, *rnmt1*-*3* and *dxo1*.**

Significantly enriched molecular functions up-/down-regulated DEGs under mock-control **(A** and **B)** and ABA-treated **(C** and **D)** conditions. Circle size represents gene counts per term. Color gradient indicates statistical significance -log10 (Pvalue) levels, ranging from high significance (red) to low (blue). MF, molecular functions.

**Figure S6. GO enrichment of BP and CC categories in DE
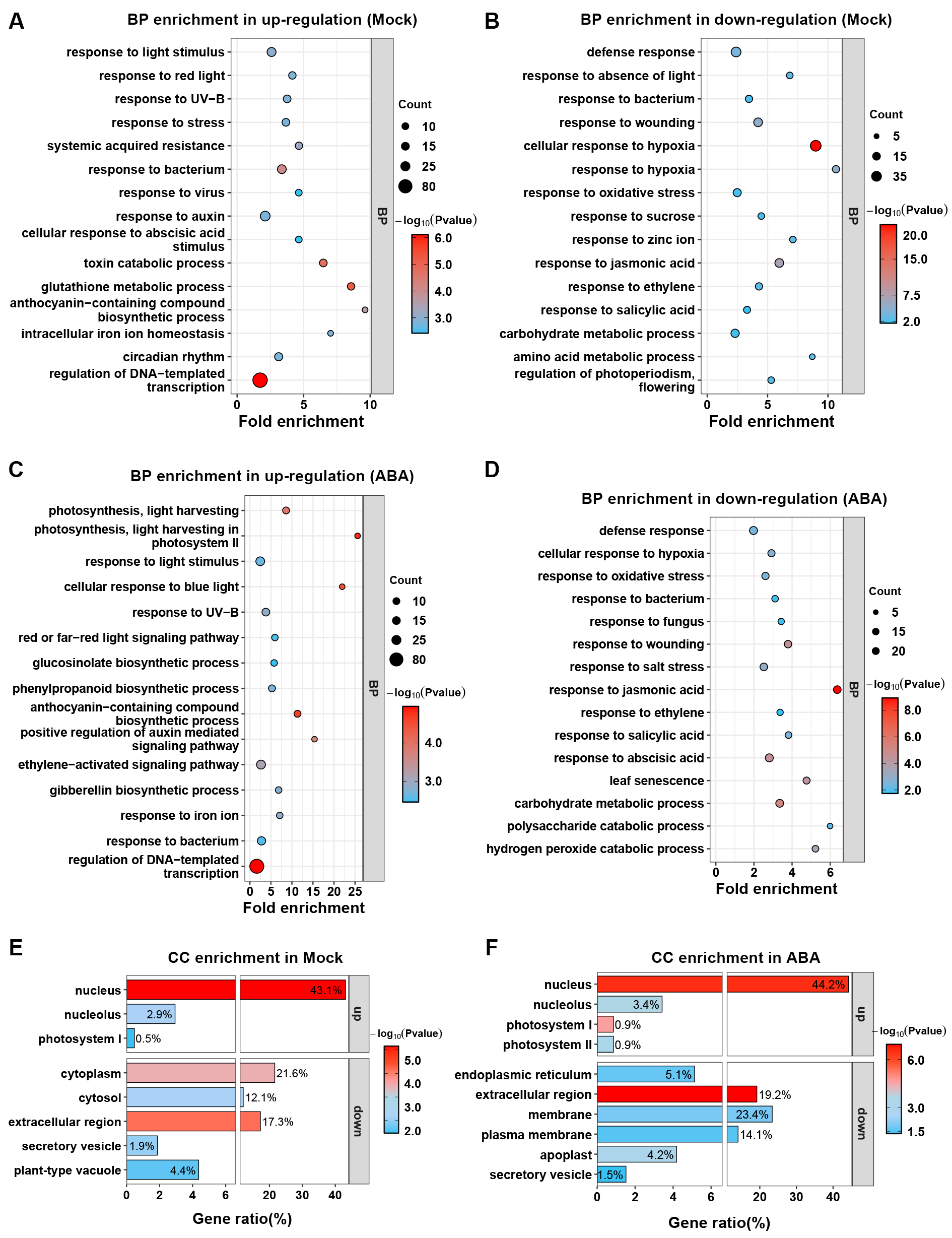
Gs of *dxo1*.**

**(A-D)** Top enriched biological processes up-/down-regulated DEGs under mock-control **(A** and **B)** and ABA-treated **(C** and **D)** conditions.

**(E and F)** Cellular component enrichment under control **(E)** and ABA-treated **(F)** conditions. Color gradient indicates statistical significance -log10 (Pvalue) levels, ranging from high significance (red) to low (blue). BP, Biological Process; CC, Cellular Compartment.

**
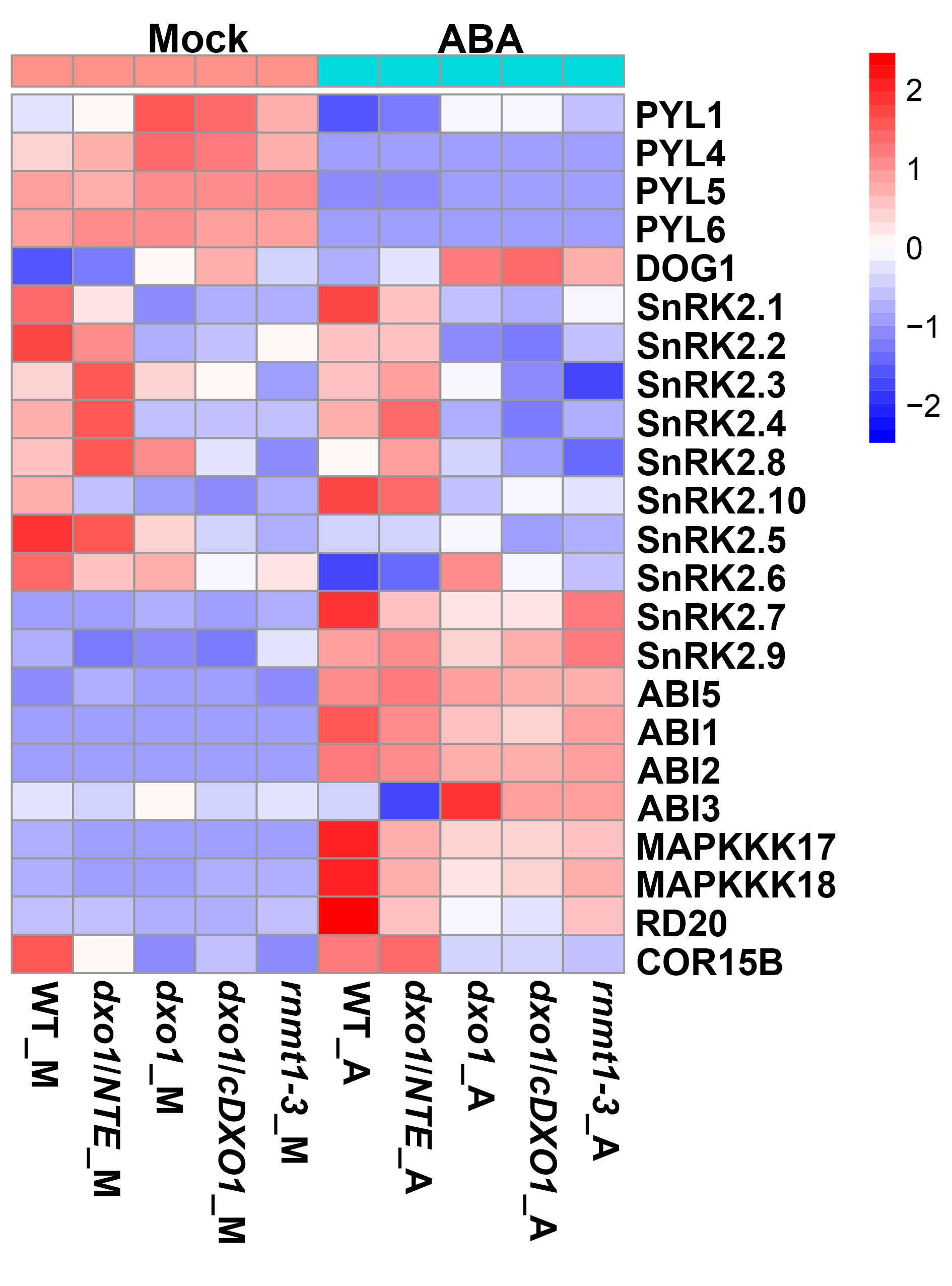
Figure S7. Expression profiles of ABA receptors, signaling transduction and responses.**

Heatmap of ABA receptor, signaling and responsive genes’ expression. The color bar indicates expression levels from high (red) to low (blue). M, mock condition; A, ABA condition.

**
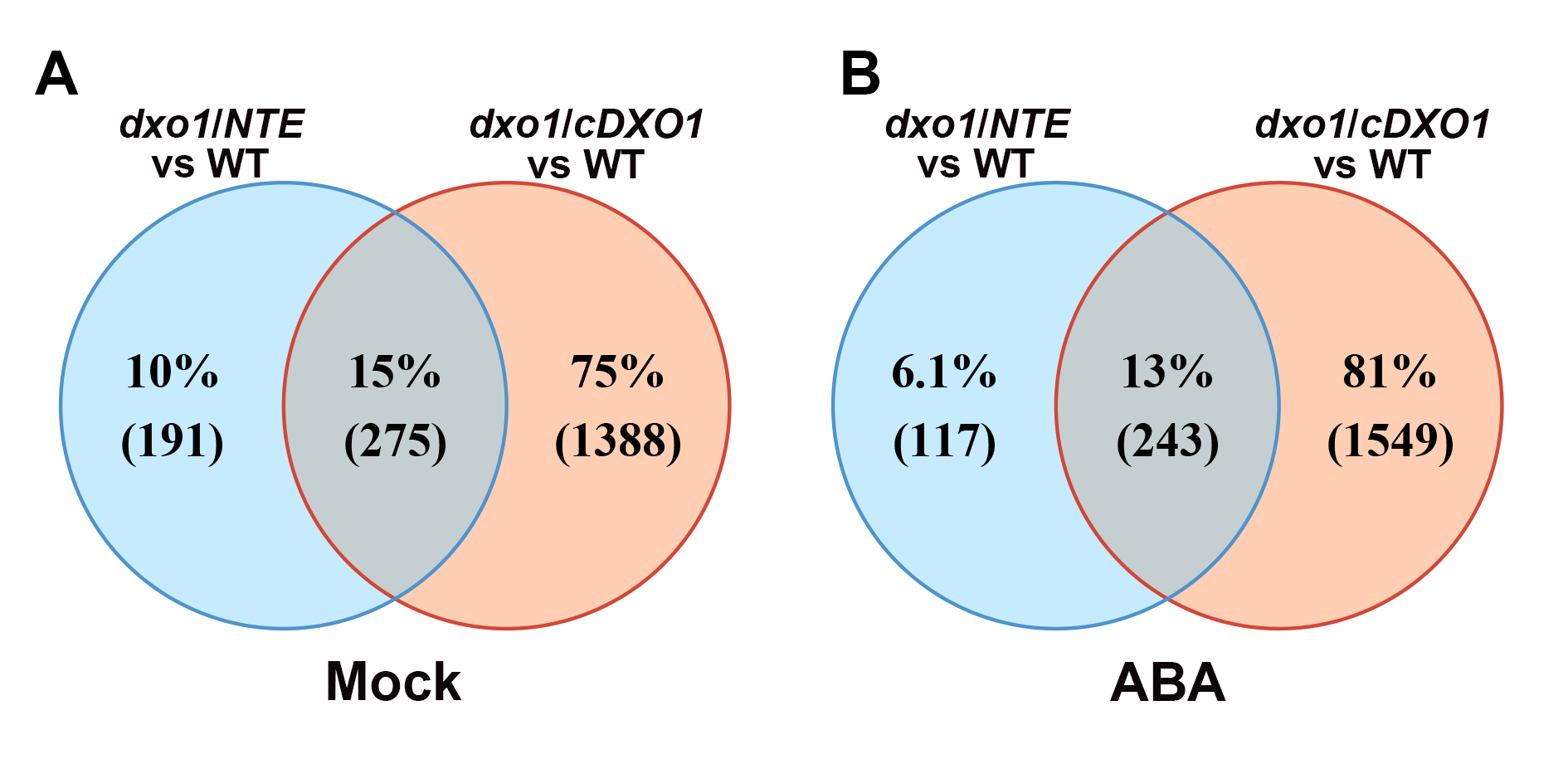
Figure S8. Overlap analysis of DEGs.**

**(A** and **B)** Venn diagram showing DEGs of dxo1/NTE-vs-WT overlaps with DEGs of dxo1/cDXO1-vs-WT under mock **(A)** and ABA **(B)** conditions.
